# Outcome and Prognostic Factors in Cats Undergoing Resection of Intestinal Adenocarcinomas: 58 Cases (2008–2020)

**DOI:** 10.3389/fvets.2022.911666

**Published:** 2022-06-27

**Authors:** Peter S. Czajkowski, Nicola M. Parry, Carrie A. Wood, Sue A. Casale, Whitney E. Phipps, Jennifer A. Mahoney, Daniel I. Spector, Lori Lyn Price, John Berg

**Affiliations:** ^1^Foster Hospital for Small Animals at Tufts Cummings School of Veterinary Medicine, North Grafton, MA, United States; ^2^Veterinary Pathology Center, University of Surrey School of Veterinary Medicine, Guildford, United Kingdom; ^3^Massachusetts Society for the Prevention of Cruelty to Animals, Angell Animal Medical Center, Boston, MA, United States; ^4^Pieper Veterinary Emergency and Specialty, Middletown, CT, United States; ^5^Matthew J. Ryan Hospital of the University of Pennsylvania, Philadelphia, PA, United States; ^6^Schwarzman Animal Medical Center, New York, NY, United States; ^7^Tufts Clinical and Translational Science Institute, Tufts University, Boston, MA, United States; ^8^Tufts Medical Center, Institute of Clinical Research and Health Policy Studies, Boston, MA, United States

**Keywords:** feline intestinal adenocarcinoma, intestinal carcinoma, oncologic surgery, carcinomatosis, feline surgery

## Abstract

The purpose of this multi-institutional retrospective study was to expand the available data pertaining to pre-operative clinical findings, progression-free and overall survival times, and potential prognostic factors for cats undergoing surgery for intestinal adenocarcinomas. Fifty-eight cats treated over a 12-year period were included in the study. Progression-free and overall survival times were estimated using Kaplan-Meier analyses. Potential prognostic variables were evaluated for associations with progression-free and overall survival using univariate Cox proportional hazards regression analyses. Prior to surgery, the intestinal mass was identified using ultrasonography in 89% of cats in which it was applied; however, imaging findings suggestive of intrathoracic metastases were observed in only 9% of cats. Among 22 cats undergoing ultrasound-guided fine needle aspiration cytology, the results agreed with the results of histopathology in only 10 cats. Discordant results were most commonly related to the presence of marked inflammation in cytology samples, which may have obscured the presence of neoplastic cells. Diffuse intestinal small cell lymphoma was identified as a comorbidity in 5 cats. Resection of the tumor with the objective of obtaining wide surgical margins was performed in each cat. On histopathology, 20 tumors were classified as mucinous adenocarcinoma and 28 were adenocarcinoma not otherwise specified. Intestinal transection site margins were complete in 94% of cats; however, complete mural margins were present in only 15% of cats. Local lymph node metastases were identified in 52% of cats and carcinomatosis was diagnosed in 81% of cats. Disease progression was documented in 32 of the 58 cats (55%). Of these 32 cats, 14 (43%) had local recurrence of the primary intestinal tumor. Median progression-free survival was 203 days (95% CI 130–299 days), and median overall survival time was 284 days (95% CI 200–363 days). Mitotic count was inversely associated with progression-free survival (HR 1.04; 95% CI 1.01–1.07, *P* = 0.005); however, none of the remaining potential prognostic factors, including administration of adjuvant chemotherapy, were significantly associated with progression-free or overall survival. Feline intestinal adenocarcinoma remains an aggressive and highly fatal disease. Large, randomized controlled clinical trials will be needed to improve the survival prospects for affected cats.

## Introduction

Intestinal adenocarcinoma is the second most common alimentary neoplasm in cats, following lymphoma, and accounts for one-third of all feline intestinal tumors (1, 2). The disease frequently develops in the small intestine, although in one report ~70% of tumors developed in the colon (1). Rates of local and distant metastases have been reported to range from 55 to 76 percent (3–7). Wide surgical resection with or without adjuvant chemotherapy is currently the primary mode of therapy (2, 8, 9). Existing retrospective studies reporting on prognostic factors and survival times describe relatively small populations of cats, but consistently suggest that outcomes are highly variable and that prolonged survival times are occasionally observed in the presence of systemic disease (4, 6, 7, 10).

The earliest available studies of long-term outcome in cats undergoing surgery for intestinal adenocarcinomas were published in the 1980's and were limited by perioperative mortality rates of up to 48% (4). The first of these studies reported a median survival time (MST) of only 5 weeks among 19 cats undergoing surgery (3); however, in a subsequent study, the mean survival time among 11 cats treated surgically was 15 months (4), suggesting that cats with advanced disease may experience prolonged survival. Two more recent studies have investigated outcome in 21 (5) and 18 (7) cats undergoing surgery for colonic adenocarcinomas. In the first of these studies (5), 5 cats underwent subtotal colectomy as opposed to less aggressive surgical procedures such as marginal mass resection or incisional biopsy, and 4 cats received adjuvant doxorubicin. Approximately 36% of tumors in the study involved the cecum or ileocecocolic junction. Both subtotal colectomy and administration of doxorubicin were significantly associated with increased MST. In the subsequent study (7), 18 cats received subtotal colectomy and adjuvant chemotherapy with carboplatin. In this study, 50% of tumors were considered cecal or ileocecal. The MST was 269 days, and presence of metastasis to mesenteric lymph nodes or distant sites was associated with decreased survival. Finally, in a study (6) of 18 cats with small intestinal adenocarcinoma that were either treated by resection and anastomosis or that did not undergo surgery, MST was 365 days among cats treated with surgery, and surgery was associated with improved survival (6). Presence or absence of gross metastatic disease was not found to influence survival times.

The response of feline intestinal adenocarcinoma to treatment appears to be influenced by a variety of factors, and providing accurate guidance to owners who are considering surgery remains challenging (5–7, 11). The purpose of the present multi-institutional retrospective study was to expand the available data pertaining to pre-operative clinical findings, progression-free and overall survival times, and prognostic factors for cats undergoing surgery for intestinal adenocarcinomas.

## Materials and Methods

### Medical Records Review

The medical record databases of the reporting veterinary referral centers (Foster Hospital for Small Animals at Tufts Cummings School of Veterinary Medicine, MSPCA Angell Animal Medical Center, Pieper Veterinary Emergency and Specialty, Matthew J. Ryan Veterinary Hospital of the University of Pennsylvania, and the Schwarzman Animal Medical Center) were searched for cats with intestinal tumors diagnosed via cytology or histopathology as carcinomas or adenocarcinomas between January 1, 2008 and December 31, 2020. Inclusion criteria were a histopathologic confirmation of intestinal adenocarcinoma, surgical resection of the primary tumor, and availability of follow-up information. Cats that survived fewer than 14 days after surgery were considered to have had surgical complication-related deaths and were excluded.

### Data Collection

Data collected from electronic medical records included signalment, presenting clinical signs and their duration, physical examination findings, results of pertinent bloodwork, diagnostic imaging findings, surgical findings, histologic or cytologic reports, and chemotherapy protocols. Outcome data included time until development of progressive disease, when applicable, and date and cause of death. Progression-free intervals and survival times were measured from the day of surgery. In cats with documented progressive disease prior to euthanasia or death, the cause of death was presumed to be tumor-related if no other cause of death was specified in the medical record.

### Histopathology Review

All available hematoxylin and eosin (HE)-stained slides were reviewed by one board-certified veterinary anatomic pathologist (NMP). When slides were unavailable, the diagnosis was based on the initial histologic or cytologic reports and the pathologist was blinded to these reports. Tumors were histologically evaluated and classified according to the predominant histologic subtype, based on World Health Organization (WHO) criteria described for tumors of the colon in humans (12) as has been reported in previous feline intestinal tumor literature (13, 14). Tumors were also categorized according to degree of glandular differentiation (well-differentiated or poorly-differentiated) (12). Additional histologic parameters assessed include mitotic count, lymphatic and vascular invasion, and lymph node metastasis.

Histologic assessments of surgical margins were performed at 3 sites within each resected specimen: at the 2 intestinal transection sites and at the intestinal wall at the site of the primary tumor (the mural margin). When slides containing the intestinal transection sites were not available for review, evaluation of transection sites was based on the original histopathologic report at the participating institution. Slides containing the primary tumor were reviewed by the pathologist (NMP) for bowel wall integrity, and an incomplete mural margin was diagnosed when penetration of tumor cells through bowel wall serosa was identified. Carcinomatosis was defined as microscopic evidence of seeding of neoplastic epithelial cells on any visceral or parietal peritoneal surface regardless of proximity to the intestinal tumor.

### Statistical Analysis

Median progression-free and overall survival times were determined using Kaplan-Meier analyses. Cats were censored from the progression-free analysis if they were lost to follow-up with no evidence of tumor recurrence, if they died or were euthanized due to non-tumor related causes, or if they were alive with no evidence of tumor recurrence at the time of most recent follow-up. Disease progression was documented using thoracic radiography, abdominal ultrasonography, and/or abdominal palpation. Progression was also assumed to be present in cats that developed recurrence of their pre-operative clinical signs, and the cause of death was presumed to be tumor-related if no other cause was specified in the medical record for these cases. Cats were censored from the tumor-related outcome if they died from reasons unrelated to the tumor. Cats with concurrent small cell alimentary lymphoma were not censored as previous studies have indicated that these cats have a prolonged median survival time and low risk of acute death (15, 16) as compared to cats with intestinal adenocarcinoma. Univariate Cox proportional hazards analyses were used to evaluate associations of potential prognostic factors with progression-free and overall survival. To reduce the likelihood of erroneously attributing significance to chance associations, only variables that the authors believed might reasonably be expected to be associated with long-term outcome were included in the analyses. The *a priori* selected variables are shown in Tables 1, 2. Statistical significance was set at *P* ≤ 0.05. Univariate Cox regression analyses were checked for the proportional hazards assumption. Adjuvant chemotherapy was modeled as a time-dependent variable as it occurred post-baseline. Analyses were performed using commercially available software [SAS Institute Inc., 9.4 (Cary, NC)].

**Table 1:** Descriptive statistics and results of cox proportional hazards regression for progression-free and overall survival associated with categorical variables.

**Categorical variable**	**Number cats with value for variable**	**Number yes (%)**	**HR (95%CI), progression-free survival**	***P*** **-value**	**HR (95% CI), overall survival**	***P*** **-value**
Pre-operative weight loss	58	46 (79.3)	1.19 (0.52, 2.72)	0.68	1.45 (0.60, 3.50)	0.41
Concurrent small cell lymphoma	58	5 (8.6)	0.61 (0.19, 1.99)	0.41	0.72 (0.22, 2.38)	0.59
Mass identified on Abdominal ultrasound	55	49 (89.1)	2.37 (0.72, 7.80)	0.16	2.80 (0.84, 9.32)	0.09
Obstructive imaging pattern	58	15 (25.9)	1.08 (0.52, 2.23)	0.83	1.14 (0.55, 2.39)	0.72
Intrathoracic metastatic disease	43	4 (9.3)	0.93 (0.22, 3.97)	0.92	0.30 (0.04, 2.26)	0.25
Tumor location: small intestine^a^	60	15 (25.0)	0.76 (0.36, 1.61)	0.47	0.86 (0.41, 1.80)	0.69
Tumor location: ileocecocolic junction^a^	60	20 (33.3)	b	b	0.96 (0.48, 1.93)	0.91
Tumor location: large intestine^a^	60	25 (41.7)	b	b	1.13 (0.58, 2.21)	0.71
Lymph node metastasis	44	23 (52.3)	1.91 (0.87, 4.17)	0.10	1.74 (0.81, 3.74)	0.16
Lymphatic or venous invasion	47	4 (8.5)	0.92 (0.28, 3.08)	0.89	0.70 (0.17, 2.98)	0.63
Carcinomatosis	48	39 (81.3)	1.48 (0.61, 3.63)	0.39	1.25 (0.51, 3.05)	0.63
Complete surgical margins	48	8 (16.7)	0.82 (0.31, 2.13)	0.68	1.05 (0.40, 2.75)	0.92
Tumor type: adenocarcinoma not otherwise specified (NOS)	48	28 (58.3)	0.92 (0.45, 1.86)	0.81	0.95 (0.47, 1.93)	0.89
Tumor differentiation grade	48	45 (93.8)	3.46 (0.47, 25.72)	0.22	2.77 (0.37, 20.56)	0.32
Tumor size: small (<1 cm largest diameter)	45	4 (8.9)	Reference		Reference	
Tumor size: intermediate (1–3 cm largest diameter)	45	15 (33.3)	0.79 (0.25, 2.51)	0.69	0.76 (0.2, 2.90)	0.69
Tumor size: large (>3 cm largest diameter)	45	26 (57.8)	0.99 (0.33, 2.99)	0.99	1.57 (0.46, 5.42)	0.48

a *Two cats were diagnosed with intestinal adenocarcinomas that involved 2 adjacent intestinal segments. Each cat had a tumor extending from the ileocecocolic junction to the proximal large intestine*.

*^b^The proportional hazards assumption was not met for tumor locations in large intestine and ileocecocolic junction when evaluating for progression-free survival*.

**Table 2:** Descriptive statistics and results of cox proportional hazards regression for progression-free and overall survival associated with continuous variables.

**Continuous variable**	**No. of cats with a value for the variable**	**Mean (SD)**	**HR (95% CI) progression-free survival**	***P*** **-value**	**HR (95% CI) overall survival**	***P*** **-value**
Mitotic count	52	17.65 (13.45)	1.04 (1.01, 1.07)	0.005	1.02 (1.00, 1.05)	0.08
Adjuvant chemotherapy	58	28 (48.3)	1.50 (0.80, 2.81)	0.21	1.72 (0.87, 3.40)	0.12

## Results

Eighty-seven cats that met the criteria for inclusion were identified during the review of electronic records. Twenty-nine cats were excluded (incomplete medical records, *n* = 8; lost to follow up, *n* = 7; surgical resection not attempted, *n* = 7; perioperative mortality, *n* = 5; and intraoperative euthanasia, *n* = 2), leaving a study population consisting of 58 cats (Foster Hospital for Small Animals at Tufts Cummings School of Veterinary Medicine *n* = 23, MSPCA Angell Animal Medical Center *n* = 21, Pieper Veterinary Emergency and Specialty *n* = 6, Matthew J. Ryan Veterinary Hospital of the University of Pennsylvania *n* = 6, and the Schwarzman Animal Medical Center *n* = 2). Median age at presentation was 12 years (range 6–17 years). Breeds included were domestic shorthair (*n* = 31), domestic longhair (*n* = 11), domestic medium hair (*n* = 3), Siamese (*n* = 3), Maine Coon (*n* = 2), Balinese (*n* = 1), Bengal (*n* = 1), Oriental Shorthair (*n* = 1), Tonkinese (*n* = 1), Turkish Angora (*n* = 1), and mixed breed (*n* = 1). Breed was not specified for 2 cats. Thirty-one cats were castrated males, and 27 cats were spayed females. There were no intact animals in the study.

Clinical signs at presentation included weight loss (*n* = 46), vomiting (*n* = 35), hyporexia (*n* = 28), diarrhea (*n* = 13), constipation or tenesmus (*n* = 12), and hematochezia (*n* = 6). Median duration of clinical signs was 30 days (range 1–365 days). An abdominal mass was noted on palpation in 20 cats. No characteristic hematologic or serum biochemical abnormalities were appreciated, although hematologic screening was not standardized among cases. Neutrophilia or monocytosis, indicative of an inflammatory leukogram, was noted in 20 cats. Hypoalbuminemia was noted in 4 cats and anemia (PCV <30%) was identified in 14 cats. Diffuse small cell intestinal lymphoma was diagnosed as a comorbidity in 5 cats. Lymphoma was diagnosed months to years prior to presentation in 3 cats and was diagnosed in 2 cats during histopathology of the surgically resected adenocarcinoma.

Thoracic imaging was performed in 43 cats (42 thoracic radiographs, 1 thoracic CT). Findings suggestive of intrathoracic metastatic disease were documented in 4 cats (9%). Pulmonary nodules were noted in 1 cat, a cranial mediastinal mass was noted in 1 cat, and sternal lymphadenopathy was identified in 2 cats. Abdominal ultrasonography was performed preoperatively in 55 cats and revealed an intestinal mass in 49 cats (89%); evidence of intestinal obstruction in 15 cats (27%); and varying amounts of free peritoneal fluid in 9 cats (16%). Ultrasound-guided fine needle aspiration of the intestinal mass was performed in 22 cats; however, the cytologic and histopathologic diagnoses agreed in only 10 of these cats (45%). The most common cause of disagreement was the presence of significant inflammation in cytology samples, which may have obscured the presence of malignant cells.

Resection of the intestinal tumor with the objective of obtaining wide surgical margins was performed in each of the 58 cats. Two cats had tumors involving adjacent intestinal segments, creating a total of 60 intestinal tumor locations. In both cats, tumors extended from ileocecocolic junction into the large intestine. Tumor size was reported in 45 cases, and median maximal diameter was 2.5 cm (range 0.5–8 cm). Twenty tumors were diagnosed as mucinous adenocarcinoma and 28 tumors were diagnosed as adenocarcinoma not otherwise specified (NOS). No other WHO histologic subtype was identified. Forty-five tumors were well-differentiated while three were poorly-differentiated. Median mitotic count was 15 mitotic figures (range 2–64) in an area of 2.37 mm^2^ [representing 10 high-power fields evaluated with a 40x objective, 22 mm ocular field number, 0.55 mm field of view diameter (17)]. Lymphatic or vascular invasion was identified in 4 cats. Draining lymph nodes were evaluated in 44 cats and metastasis was identified in 23 cats (52%).

Histologic margin assessments at intestinal transection sites were performed in 52 cats during initial histologic evaluation at the participating institutions. Intestinal transection site margins were histologically complete in 49 cats (94%) and incomplete in 3 cats (6%). Sectioned slides were available for review by the pathologist (NMP) in only 48 of these cases. Complete mural margins (i.e., lack of transmural invasion by the primary tumor) were identified in only 15% of cats. Carcinomatosis was identified in 39 cats (81%). Of the 39 cats with carcinomatosis, transmural invasion of the primary tumor was seen in 38 cats. An additional 3 cats were diagnosed with transmural invasion without microscopic peritoneal seeding of tumor cells (i.e., breach of serosa without carcinomatosis) in the slides available for review.

At least one dose of chemotherapy was given to 28 cats. Chemotherapy dosages and protocols were at the discretion of the oncologists at each participating institution. Protocols included carboplatin (*n* = 21), doxorubicin (*n* = 11), toceranib phosphate (*n* = 9), mitoxantrone (*n* = 4), chlorambucil (*n* = 5), cyclophosphamide (*n* = 3), lomustine (*n* = 2), and masitinib (*n* = 1).

**Figure 1:**
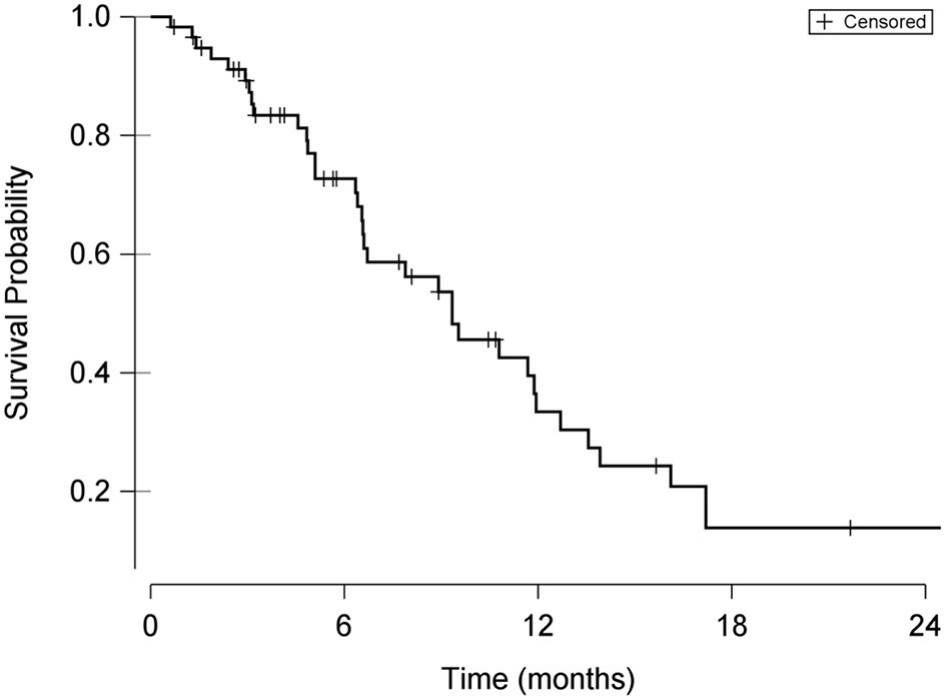
Kaplan-Meier curve demonstrating overall survival probability from date of surgery in cats undergoing surgical resection of intestinal adenocarcinoma. Hash marks in the curve indicate times of censoring.

Disease progression was documented through either restaging abdominal ultrasound or physical examination findings (palpation of mid-abdominal mass) in 32/58 cases (55%). Disease progression was suspected based on recurrence of presenting clinical signs in an additional 8 cats. Local recurrence of the primary intestinal tumor was identified on imaging in 14 of the 32 cats (43%) with disease progression. A Kaplan-Meier plot of overall survival for the 58 cats included in the study is shown in Figure 1. The median progression-free survival time was 203 days (95% CI 130–299 days) and the median survival time was 284 days (95% CI 200–363 days).

Descriptive statistics for the potential prognostic factors selected for Cox proportional hazards regression are shown in Table 1 (categorical variables) and Table 2 (continuous variables). Mitotic count was significantly (*P* = 0.005) inversely associated with progression-free survival (HR 1.04; 95% CI 1.01–1.07); however, none of the remaining potential prognostic factors selected were significantly associated with overall survival.

## Discussion

The primary aim of this study was to expand the available literature concerning the clinical presentation, diagnostic findings and outcomes for cats undergoing surgical resection of intestinal adenocarcinomas.

Previous reports have suggested that Siamese cats may be over-represented among affected cats (1, 3, 18, 19), and our study population, which contained 3 Siamese cats and 1 each of 5 other Asian breeds, appeared to be consistent with this. Overall, Asian breeds accounted for 14% of the study population.

Clinical signs were generally typical of those expected in cats with severe chronic intestinal obstruction, and as a result, ~35% of the tumors could be detected on abdominal palpation. Abdominal ultrasound proved to be a highly sensitive imaging test for identifying the primary tumor, confirming the presence of an intestinal mass in 89% of the 55 cats in which it was performed. However, among 22 cats undergoing ultrasound-guided fine needle aspiration cytology, the results agreed with the results of histopathology of the resected tumor specimens in only 10 cats. Discordant results were frequently related to the presence of marked inflammation in cytology samples, which may have obscured the presence of neoplastic cells. This limitation of cytology in the diagnosis of feline intestinal adenocarcinomas has been reported previously (19).

On histopathology, all primary tumors were diagnosed as either mucinous adenocarcinoma or adenocarcinoma not otherwise specified (NOS). The majority of tumors were considered to be well-differentiated. Although lymphatic or vascular invasion of the primary tumors was unusual, metastases to mesenteric lymph nodes were observed on histopathology in 23 of 44 cats.

Histopathologic assessment of surgical margins was complicated by the complex geometry of the resection samples and by the involvement of multiple institutions with variable approaches to margin analysis. An effort was made to standardize the analysis by obtaining the tissue to be assessed from the same 3 locations in each resection sample: at one or both intestinal transection sites and through the intestinal wall at the site of the tumor. Invasion through the wall of the tumor at the serosal surface was considered to constitute an incomplete margin. Not surprisingly, transection site margins were complete in a large majority of cats, indicating that surgeons were aggressive in removing an adequate length of intestine on either side of the tumor. However, mural margins were frequently incomplete, indicating transmural invasion by the tumor and leading to a high rate of carcinomatosis. A complicating factor during margin analyses is shrinkage of normal tissues adjacent to the tumor due to exposure to formalin during the delay between surgery and histopathologic tissue assessment. Previous studies have shown contraction of canine intestinal tissue by up to 26.3% within 24 h of formalin fixation (20), and similar findings have been reported in the human literature (21). Shrinkage of normal tissues adjacent to the tumors may have falsely increased the rate of incomplete margins in the present study.

The high rate of carcinomatosis observed in the study was likely related to our criteria for identifying the condition. Carcinomatosis was considered to be present if microscopic seeding of neoplastic cells was noted on any peritoneal surface, irrespective of proximity to the primary tumor. In all cats with carcinomatosis, seeding was noted on the peritoneal surface immediately adjacent to the primary tumor.

The disease-free and overall MST's observed in the study were consistent with those reported previously (4–7). Feline intestinal adenocarcinoma has consistently proven to be an aggressive neoplasm with high rates of dissemination via metastases or carcinomatosis evident at the time of diagnosis (4, 5, 7, 18). Our findings were consistent with this: 52% of cats undergoing mesenteric lymph node biopsy at the time of surgery for the primary tumor had nodal metastases, and 81% of cats were diagnosed with carcinomatosis. Eventual disease progression was documented in 55% of cats, and local recurrence of the primary tumor was observed in 43% of cats.

Administration of adjuvant chemotherapy was not significantly associated with improved disease-free or overall survival, although it is possible that a selection bias was present, causing owners to be most likely to elect chemotherapy for cats that they perceived to have advanced disease. Additionally, the chemotherapy protocols used in the study were at the discretion of the attending oncologist at participating institutions, and the lack of chemotherapy standardization markedly limited our ability to assess its efficacy.

The only variable found to be statistically associated with progression-free or overall survival was the mitotic count within resected tumor specimens, with lower mitotic counts correlating with better progression-free survival. Mitotic count is an indicator of cellular proliferation rates and tumor aggressiveness, and is a frequent component of tumor grading systems (17). Mitotic counts are easy and economical for pathologists to perform, and methods for improving their utility by standardizing techniques are evolving (17).

The present study had many of the limitations that are common to multi-institutional, non-randomized studies. Several aspects of diagnostics, tumor staging, surgical and medical treatment and monitoring were not standardized among institutions. The study population was small, and most participating institutions were not able to provide all of the information requested during data collection. Disease progression was considered to have occurred in cats with recurrence of clinical signs whether or not repeat imaging was available. This method of reporting is commonly seen in feline intestinal tumor literature (22–24), although this method may have overrepresented the number of cats with progressive disease in the current study. The authors elected to not perform a multivariate analysis due to the limited number of cats with complete data sets, which could have resulted in a model with unreliable conclusions and a lack of generalizability. Of the 87 cats that met the inclusion criteria for the study, twenty-nine were excluded. A significant proportion of the cats that remained in the study were censored from the statistical analyses, further limiting the power of the study.

Feline intestinal adenocarcinoma remains a common and highly fatal disease. Thorough and frequent monitoring for progressive disease following surgery is essential, as individual cats may be affected by intrathoracic metastases, abdominal metastases, or local tumor recurrence. Large, randomized controlled clinical trials will be needed to improve the survival prospects for affected cats.

## Data Availability Statement

The raw data supporting the conclusions of this article will be made available by the authors, without undue reservation.

## Author Contributions

PC contributed to study design, data acquisition, analysis, interpretation, and manuscript drafting. NP contributed to study design, performed all histopathologic review, and manuscript review. CW contributed to study design and manuscript review. SC, WP, JM, and DS data collection and manuscript review. LP performed statistical analysis and manuscript review. JB contributed to study design, contributed significantly to manuscript drafting, and review. All authors contributed substantially to the production and review of this manuscript.

## Funding

The project described was supported by the National Center for Advancing Translational Sciences, National Institutes of Health, Award Number UL1TR002544. The content is solely the responsibility of the authors and does not necessarily represent the official views of the NIH.

## Conflict of Interest

The authors declare that the research was conducted in the absence of any commercial or financial relationships that could be construed as a potential conflict of interest.

## Publisher's Note

All claims expressed in this article are solely those of the authors and do not necessarily represent those of their affiliated organizations, or those of the publisher, the editors and the reviewers. Any product that may be evaluated in this article, or claim that may be made by its manufacturer, is not guaranteed or endorsed by the publisher.
